# Intact endogenous pain inhibition in complex regional pain syndrome type 1

**DOI:** 10.1097/PR9.0000000000001358

**Published:** 2025-11-12

**Authors:** Florin Allmendinger, Laura Sirucek, Iara De Schoenmacker, Paulina Simonne Scheuren, Florian Brunner, Petra Schweinhardt, Michèle Hubli

**Affiliations:** a Spinal Cord Injury Center, Balgrist University Hospital, University of Zurich, Zurich, Switzerland; b Department of Chiropractic Medicine, Balgrist University Hospital, University of Zurich, Zurich, Switzerland; c Center for Neuroplasticity and Pain (CNAP), Department of Health Science and Technology, Aalborg University, Aalborg, Denmark; d Biomedical Data Science Lab, Institute of Translational Medicine, Swiss Federal Institute of Technology (ETH) Zurich, Zurich, Switzerland; e International Collaboration on Repair Discoveries, University of British Columbia, Vancouver, BC, Canada; f Department of Anesthesiology, Pharmacology & Therapeutics, Faculty of Medicine, University of British Columbia, Vancouver, BC, Canada; g Physical Medicine and Rheumatology, Balgrist University Hospital, University of Zurich, Zurich, Switzerland

**Keywords:** Endogenous pain modulation, Descending pain inhibition, Complex regional pain syndrome, Conditioned pain modulation, Chronic pain

## Abstract

**Introduction:**

Impaired endogenous pain modulation is a common characteristic of various chronic pain conditions and may be a key mechanism underlying the development of chronic pain in complex regional pain syndrome (CRPS). One way to assess endogenous pain modulation in humans is through conditioned pain modulation (CPM) paradigms.

**Objectives:**

This study aimed to assess CPM capacity in chronic CRPS type 1 and its potential association with the individual pain phenotype.

**Methods:**

Sixteen individuals with chronic CRPS type 1 and 15 age- and sex-matched healthy controls (HC) were enrolled. Parallel and sequential CPM paradigms were performed at 2 testing sites: the most painful area and a remote, pain-free area using pressure pain thresholds as a test stimulus and a cold water bath as a conditioning stimulus. Individuals with CRPS underwent pain phenotyping to assess the characteristics of CRPS-related pain.

**Results:**

The CPM capacity did not differ between individuals with CRPS and HC (*P*'s > 0.05). Complex regional pain syndrome showed intact pain inhibition assessed in both areas in the parallel and sequential CPM paradigm (*P*'s < 0.05). Furthermore, the CPM capacity of individuals with CRPS did not correlate with individual pain characteristics (ie, pain intensity, pain extent, and pain duration; *P*'s > 0.05).

**Conclusion:**

Our results indicate that in individuals with chronic CRPS type 1, endogenous pain inhibition is intact and comparable with healthy individuals. Therefore, pathomechanisms underlying chronic CRPS are likely related to increased pain facilitatory rather than impaired inhibitory processes.

## Abstract 2

Supplemental Digital Content is Available in the Text.

Chronic complex regional pain syndrome exhibits intact conditioned pain modulation, suggesting the importance of impaired pain facilitatory rather than pain inhibitory processes in this chronic pain cohort.

## 1. Introduction

Complex regional pain syndrome (CRPS) is characterized by a myriad of signs and symptoms after an initial injury or surgical procedure, including persisting, disproportionate pain, hyperalgesia, and allodynia in the affected limb.^1,3,16^ The proposed pathomechanisms of CRPS are diverse and range from genetic predispositions, psychological distress, inflammatory processes, and vasomotor dysfunction to sensitization of the nociceptive system, decreased antinociceptive modulation, and spinal disinhibition.^7,10^ Specifically, alterations of the facilitatory and inhibitory balance in the endogenous pain modulatory system might contribute to observed clinical features in CRPS, such as hyperalgesia and allodynia.^6^

One experimental approach to probe this pain modulatory capacity is conditioned pain modulation (CPM),^43^ a paradigm testing the modulation of a noxious test stimulus (TS) by a second heterotopically applied noxious conditioning stimulus (CS).^44^ Various studies have reported a lack of pain inhibition in diverse chronic pain conditions,^22^ and lower CPM efficiency has been linked to a higher risk of developing chronic pain.^45^ However, investigations of endogenous pain modulation in CRPS are sparse. To the best of our knowledge, there are only 2 studies which investigated the endogenous pain modulatory system in individuals with CRPS,^20,38^ using different experimental approaches.

Using a CPM paradigm, *Kumowski et al., 2017*, found intact endogenous pain inhibition in CRPS, potentially attributed to the fact that enrolled patients were still in an early stage of the disease (duration < 1 year), where maladaptive changes of the central nervous system are less pronounced than in more progressed stages of CRPS.^4^ By contrast, *Seifert* et al.*, 2009*, found decreased adaptation to repetitive painful electrical stimulation in both the affected and the unaffected arm of individuals with CRPS compared with healthy participants. Furthermore, after repetitive electrical stimulation, individuals with CRPS developed larger areas of pinprick hyperalgesia on their affected arm than healthy participants. This finding was discussed as a decrease in pain inhibitory capacity and a shift from pain inhibition toward facilitation in CRPS. The contrasting findings of these 2 studies, despite CPM and pain adaptation both representing aspects of endogenous pain modulation,^41^ highlight that our understanding of alterations in pain inhibition and facilitation in CRPS is incomplete and warrants further research.

Therefore, the first aim of this study was to investigate the pain modulatory capacity in chronic CRPS using a CPM paradigm. The second aim was to explore the debated relationship between CPM capacity and the individual pain phenotype (ie, pain intensity, pain duration, and spatial pain extent),^9^ as well as psychological factors (ie, anxiety, depression, and pain catastrophizing).^28^ Conditioned pain modulation was investigated in the CRPS-affected, painful limb as well as a remote, pain-free area to disentangle potential generalized and additional segmental pain facilitatory effects.

## 2. Methods

### 2.1. Study participants

This study was part of a larger study (Clinical Research Priority Program Pain, https://www.crpp-pain.uzh.ch/en.html) including patient cohorts with CRPS, chronic low back pain, and neuropathic pain after spinal cord injury, as well as healthy controls (HC). Participants were recruited between November 2019 and April 2022. This article includes data of individuals with CRPS type 1 (N = 16) and age- and sex-matched HC (N = 15). Individuals with CRPS were recruited at the Department of Physical Medicine and Rheumatology of the Balgrist University Hospital in Zurich, Switzerland. Inclusion criteria for individuals with CRPS were fulfillment of the Budapest Criteria,^16^ examined by a clinician with CRPS expertise (F.B.), diagnosis of CRPS type 1, and age between 18 and 80 years. Exclusion criteria were pregnancy, neurological disorders, psychiatric disorders, or history of chronic pain other than CRPS-related pain. The same exclusion criteria were applied to HC with addition of no acute pain or intake of pain medication. Written informed consent was obtained from all participants before study inclusion. The study was approved by the local ethics committee “Kantonale Ethikkommission Zürich” (EK-04/2006, PB_2016-02051) and conducted in accordance with the Declaration of Helsinki.

### 2.2. Study protocol

**Figure 1.**
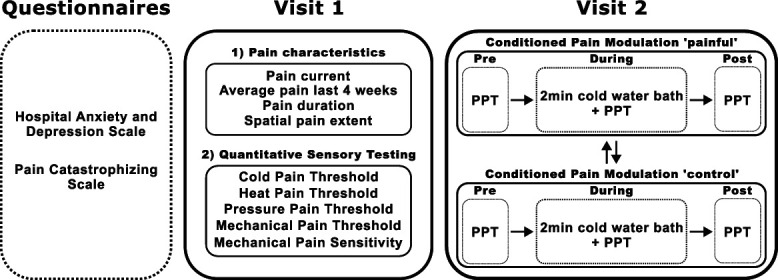
Study protocol and analyzed measurements. PPT, pressure pain threshold.

The study consisted of 2 visits comprising the evaluation of pain characteristics, psychological questionnaires, quantitative sensory testing (QST), neurophysiological assessments, and experimental pain paradigms. This article focuses on pain characteristics, psychological questionnaires (Hospital Anxiety and Depression Scale [HADS]^39^ and Pain Catastrophizing Scale [PCS]^40^), QST, and CPM paradigm. Pain characteristics and QST were assessed on visit 1, and the CPM paradigm was performed on visit 2 (Fig. 1). Both tests, QST and CPM paradigm, followed the acquisition of pain-related evoked potentials (not discussed in this article) and were performed in the most painful (hereinafter “painful”) and a remote, pain-free (hereinafter “control”) area of individuals with CRPS. The control area was defined as the contralateral, pain-free shoulder, if the painful area was an upper extremity, or the contralateral, pain-free hand, if the painful area was a lower extremity. Healthy controls were assessed in the same 2 areas as their age- and sex-matched counterparts.

### 2.3. Pain characteristics

Evaluation of pain characteristics included recording of the pain intensity on a numeric rating scale (NRS) on the day of participation and average pain intensity over the past 4 weeks, the spatial pain extent of CRPS-related pain, as well as the intake of regular pain medication. To determine the spatial pain extent, individuals with CRPS marked their painful areas on standardized body charts (including dorsal and frontal view). The recorded body charts were then scanned, digitized, and run through a custom-made algorithm, calculating the percentage of marked pixels compared with the total amount of pixels of the full body.^36^

### 2.4. Quantitative sensory testing

The QST protocol was performed as defined by the German Research Network on Neuropathic Pain (DFNS).^35^ The complete protocol, consisting of cold detection threshold, warm detection threshold, thermal sensory limen (TSL, including paradoxical heat sensation), cold pain threshold (CPT), heat pain threshold (HPT), mechanical detection threshold, mechanical pain threshold (MPT), stimulus–response function (SR function, including mechanical pain sensitivity [MPS] and dynamic mechanical allodynia), wind-up ratio (WUR), vibration detection threshold, and pressure pain threshold (PPT), was assessed in the painful area. Only a subset of the protocol (ie, CPT, HPT, MPT, SR function, WUR, and PPT) were assessed in the control area. The analysis of this article focuses only on QST parameters that can detect a gain of sensory function (ie, CPT, HPT, PPT, MPT, and MPS), indicative of possible pain facilitatory mechanisms. All participants were familiarized to each of the measurements in a pain-free area, other than the control area, before the testing. The QST data were normalized to reference values using the eQuiSTA software (version 1.3.7) provided by the DFNS and are presented as z-scores.

### 2.5. Conditioned pain modulation

The CPM paradigm consisted of the PPT as TS and the immersion of the limb contralateral to the painful limb into a cold water bath as CS. The PPT was assessed with a pressure algometer (FDN100, Wagner Instruments, USA). The temperature of the water bath was 9 ± 0.5°C, and the duration of the immersion was set to 2 minutes. The PPT was assessed before the water bath, 30 seconds after immersion of the limb, and immediately after removal of the limb from the water bath. The order of the CPM assessment of the painful and the control limb was randomized and separated by at least 10 minutes to reduce carry-over effects. Similar to the QST data, PPTs acquired during the CPM paradigm were transformed to z-scores using the eQuiSTA software.

Both the parallel and sequential CPM capacity were calculated.^44^ The parallel CPM capacity was calculated as the change of the PPT measured during the water bath compared with the PPT measured before the water bath (Equation 1). Similarly, the sequential CPM capacity was calculated as the change of the PPT measured after the water bath compared with the PPT measured before the water bath (Equation 2). Therefore, negative values represent an inhibitory effect, while positive values represent a facilitatory effect.

(1) Parallel CPM capacity=PPT during−PPT pre

(2) Sequential CPM capacity=PPT post−PPT pre

In addition, all participants were classified as CPM-inhibitors, CPM-facilitators, or CPM-nonresponders based on the standard error of measurement (SEM) of the PPT z-score.^23^ The SEM was calculated using the following formula (Equation 3):

(3) $$\text{SEM}=\text{SD}\times \sqrt{\left(1-\text{ICC}\right)}$$

The standard deviation (SD) and the intraclass correlation coefficient (ICC) were calculated based on the PPT measured before the cold water bath and the PPT measured before a sham water bath (used in another part of the larger study, only in the control area)^24^ in HC. Participants showing a CPM effect larger than ±SEM were classified as CPM-inhibitors (<SEM) or facilitators (>SEM), while participants showing a smaller effect were classified as CPM-nonresponders.

### 2.6. Statistical analysis

Statistical analyses were performed using R (R version 4.2.2 for Windows). All data were tested for normality by Shapiro–Wilk tests and histograms. Fulfillment of model criteria were checked with diagnostic plots (ie, quantile-quantile plots and histograms). Statistical tests were performed at an α level of 0.05. All *P*-values were adjusted for multiple comparisons using the Benjamini–Hochberg method.

#### 2.6.1. Demographics and questionnaires

Age, height, weight, and questionnaire outcomes (ie, HADS and PCS scores) were compared between individuals with CRPS and HC using unpaired *t*-tests or Wilcoxon rank-sum tests depending on normality of the data distribution.

#### 2.6.2. Quantitative sensory testing and conditioned pain modulation

Separate linear mixed models (“lmer” function of the R package “lme4”) were used to assess the main effect of “cohort” (CRPS and HC) and “area” (painful and control) on the particular QST z-scores (CPT, HPT, PPT, MPT, and MPS) and CPM capacity (parallel and sequential). For each dependent outcome variable, 2 candidate models were evaluated: one including the main effects (“cohort” and “area”) and one also including the interaction term (“cohort × area”). The random effect “subject” was included in all models. To determine whether adding the interaction term improved the model fit, we compared the models using a likelihood ratio test (“anova” function of the R package “stats”). Interaction terms were only included in the final model if they improved the model fit. Post hoc multiple comparisons were performed on included interactions “cohort × area.” Unpaired *t*-tests were used to detect potential differences between the 2 cohorts within a tested area (eg, control area in CRPS vs control area in HC) and paired *t* test to detect differences between the 2 tested areas within a cohort (eg, painful vs control area in CRPS). In addition, one-sided *t*-tests against zero were used to assess whether a general inhibitory CPM capacity could be detected on a group level.

#### 2.6.3. Association of conditioned pain modulation effect with complex regional pain syndrome characteristics and psychological factors

Spearman correlation analyses were used to test the association of the parallel CPM effect in the control area with (1) current pain intensity, (2) average pain intensity during the past 4 weeks, (3) pain duration, (4) spatial pain extent, (5) HADS anxiety, (6) HADS depression, and (7) PCS of individuals with CRPS. The CPM effect of the control area was chosen for these correlation analyses because it is less likely to be influenced by potential pathological peripheral processes, but mainly depicting systemic endogenous pain modulatory capacity.

## 3. Results

### 3.1. Demographics and pain characteristics

**Figure 2.**
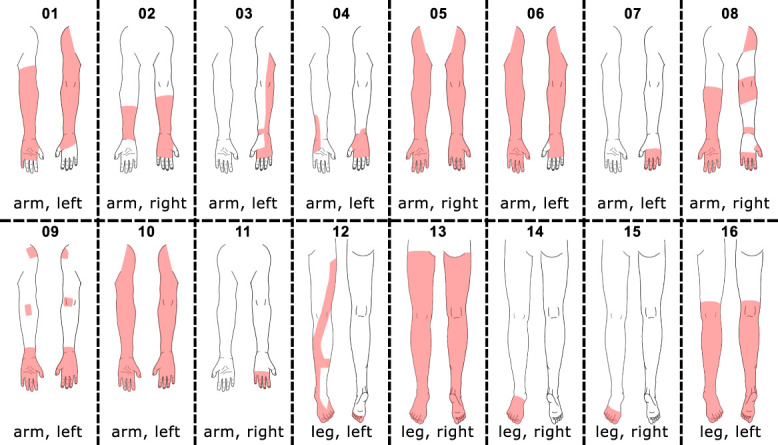
CRPS-specific pain extent (red area) within the painful limb of individuals with CRPS. The painful limb is presented in frontal (left) and dorsal (right) view. CRPS, complex regional pain syndrome.

Demographics and questionnaire outcomes of the study population are summarized in Table 1. While there were no differences in the general demographics (ie, age, height, and weight) between the cohorts, individuals with CRPS presented with higher scores for HADS anxiety, HADS depression, and PCS than HC. Complex regional pain syndrome–specific characteristics are summarized in Table 2. The CRPS-specific pain extent within the painful limb is shown in Figure 2.

**Table 1:** Demographics and questionnaire outcomes.

	CRPS, N = 16	HC, N = 15	Test value	*P*	Cohen d
Age (y)	44 ± 12	41 ± 13	W = 142.5	0.41	0.26
Height (cm)	169 ± 6	168 ± 7	t = 0.48	0.66	0.17
Weight (kg)	71 ± 14	65 ± 11	t = 1.36	0.27	0.49
HADS anxiety (score)	7.5 ± 4.0	3.7 ± 2.9	t = 3.08	0.01	1.10
HADS depression (score)	6.5 ± 5.5	1.1 ± 0.9	W = 199.5	0.006	1.35
PCS (score)	21.1 ± 12.1	6.3 ± 8.0	W = 202.5	0.005	1.44

Values are reported as mean and standard deviation.

CRPS, complex regional pain syndrome; HC, healthy controls; HADS, Hospital Anxiety And Depression Scale; PCS, Pain Catastrophizing Scale.

**Table 2:** Complex regional pain syndrome characteristics and medication intake of individuals with complex regional pain syndrome.

Pain characteristics	Mean ± SD (range)
Pain current (NRS)	4.9 ± 2.6 (1-9)
Average pain past 4 wk (NRS)	5.3 ± 2.5 (1-10)
Pain duration (mo)	34 ± 26 (6-96)
Spatial pain extent (% body surface)	6.4 ± 5.9 (0.5-18.3)

Medication	N (%)
NSAID	7 (44)
SSNRI	2 (13)
Opioids	5 (31)
Antidepressants	4 (25)
Pregabalin	3 (19)
Gabapentin	2 (13)

CRPS, complex regional pain syndrome; NRS, numeric rating scale; NSAID, nonsteroidal anti-inflammatory drugs; SSNRI, selective serotonin reuptake inhibitors.

### 3.2. Quantitative sensory testing

**Figure 3.**
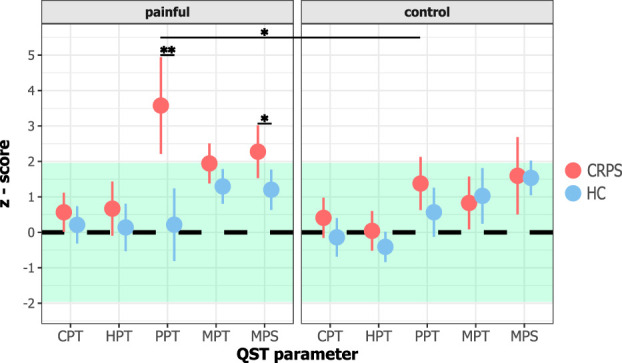
QST z-scores (mean ± SD) of the cold pain threshold (CPT), heat pain threshold (HPT), pressure pain threshold (PPT), mechanical pain threshold (MPT), and mechanical pain sensitivity (MPS) of the painful and the control area in individuals with CRPS and HC. The green area represents ±1.96 SD from normative values. *P < 0.05; **P < 0.01 (*t* test between cohorts and within cohort). CRPS, complex regional pain syndrome; HC, healthy controls; QST, quantitative sensory testing.

Quantitative sensory testing showed mechanical hyperalgesia in the painful limb of individuals with CRPS. Specifically, the PPT z-scores differed between the 2 cohorts and tested areas (“cohort × area”; F = 12.44; *P* = 0.001). Post hoc comparisons showed that PPT z-scores of the painful limb were higher in CRPS (3.58 ± 2.46) compared with HC (0.22 ± 1.85; t = 4.23; *P* = 0.004; Cohen d = 1.54) (Fig. 3). In addition, PPT z-scores of the CRPS-affected limb were increased compared with their control area (1.38 ± 1.35; t = 3.48; *P* = 0.01; Cohen d = 1.02). For MPS, the interaction effect (“cohort × area”) did not reach significance (F = 4.01; *P* = 0.054). However, the model with the interaction effect included was significantly better than the model without the interaction effect, and thus, post hoc comparisons were performed (see the Methods section “Statistical analysis”). Post hoc analysis showed that individuals with CRPS presented with higher MPS compared with HC in their painful limb (CRPS: 2.28 ± 1.40; HC: 1.20 ± 1.03; t = 2.45; *P* = 0.04; Cohen d = 0.87), but not in the control area (CRPS: 1.60 ± 1.97; HC: 1.54 ± 0.88; t = 0.11; *P* = 0.92; Cohen d = 0.04).

### 3.3. Conditioned pain modulation

The mean temperature of the water bath was 9.2 ± 0.3°C and was perceived as painful by all participants (NRS 7.9 ± 1.9). Two individuals with CRPS did not tolerate the full CPM paradigm tested in the painful limb (ie, one did not tolerate the PPT assessment due to too much pain in the painful limb and one withdrew their hand from the water bath before 60 seconds). These 2 trials were therefore excluded from the CPM analysis.

**Figure 4.**
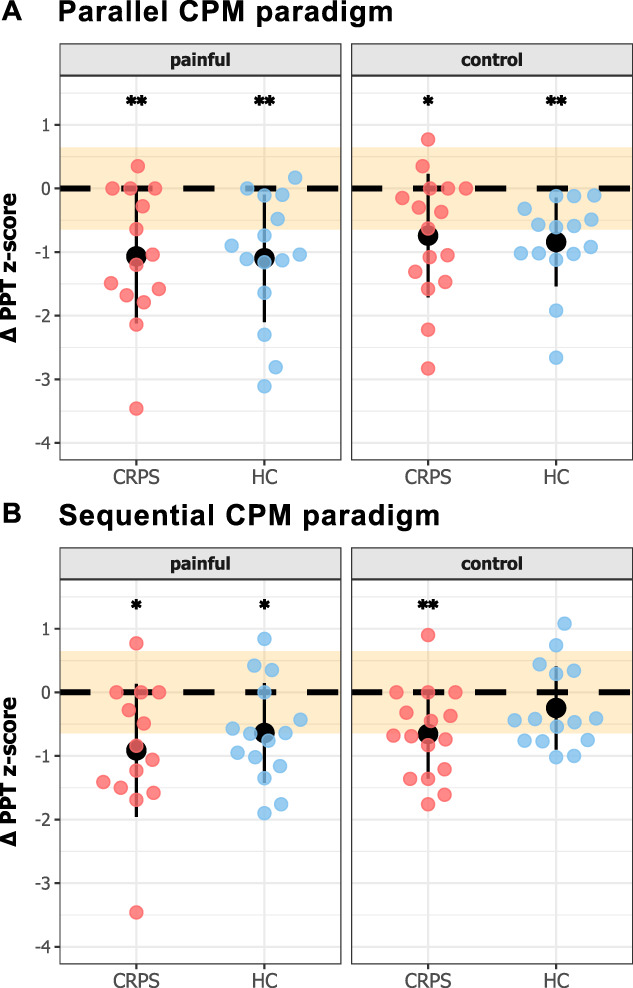
CPM effects for the painful and control area assessed in a parallel (A) and sequential design (B). CPM effect is presented as change in PPT z-score during (parallel) and after (sequential) assessment compared with the premeasurement. Negative values reflect pain inhibition, positive values pain facilitation. Black dots and lines represent group mean ± SD. The orange area represents ±SEM. CPM, conditioned pain modulation; CRPS, complex regional pain syndrome; HC, healthy controls; PPT, pressure pain threshold; SD, standard deviation; SEM, standard error of measurement. *P < 0.05; **P < 0.01 (*t* test against zero).

The CPM data are illustrated in Figure 4A (parallel) and Figure 4B (sequential). Raw values of the PPT pre, during, and post-water bath are provided in the supplementary material Table S1, http://links.lww.com/PR9/A354.

There was no significant main effect of “cohort” (parallel: F = 0.06; *P* = 0.81; sequential: F = 2.48; *P* = 0.12) or “area” (parallel: F = 1.68; *P* = 0.21; sequential: F = 2.78; *P* = 0.10) on CPM capacity, neither for the parallel nor for the sequential paradigm. This finding reveals that there were no differences in pain inhibitory capacity, tested with our CPM paradigm, between individuals with CRPS and HC. Furthermore, individuals with CRPS showed an overall inhibitory CPM capacity both in the parallel design (painful: t = −3.79; *P* = 0.005; Cohen d = −1.01; 8 inhibitors, 6 nonresponders; control: t = −3.06; *P* = 0.01; Cohen d = −0.76; 7 inhibitors, 8 nonresponders, 1 facilitator) and sequential design (painful: t = −3.27; *P* = 0.01; Cohen d = −0.87; 8 inhibitors, 5 nonresponders, 1 facilitator; control: t = −3.73; *P* = 0.005; Cohen d = −0.93; 9 inhibitors, 6 nonresponders, 1 facilitator). While the HC also showed an overall inhibitory CPM capacity tested in the parallel design (painful: t = −4.24; *P* = 0.004; Cohen d = −1.09; 10 inhibitors, 5 nonresponders; control: t = −4.67; *P* = 0.004; Cohen d = −1.21; 7 inhibitors, 8 nonresponders), when tested in sequential design, they only showed an inhibitory CPM capacity in the painful (t = −3.15; *P* = 0.01; Cohen d = −0.81; 8 inhibitors, 6 nonresponders, 1 facilitator) but not the control limb (t = −1.45; *P* = 0.14; Cohen d = −0.38; 5 inhibitors, 8 nonresponders, 2 facilitator).

### 3.4. Association of conditioned pain modulation capacity with pain characteristics

Overall, only HADS anxiety significantly correlated with the parallel CPM effect of individuals with CRPS (rho = −0.58; *P* = 0.04). Interestingly, this correlation was negative, indicating that higher levels of anxiety were associated with stronger inhibition in individuals with CRPS. Statistical trends were observed for negative correlations between CPM capacity and pain duration, HADS depression, and PCS (rho's < −0.5; *P*'s < 0.09). All results of the correlation analyses are summarized in Table 3.

**Table 3:** Spearman correlations of pain characteristics and conditioned pain modulation capacity.

	Rho	*P*
Pain current	−0.30	0.34
Average pain past month	−0.26	0.38
Pain duration	−0.55	0.053
Spatial pain extent	0.25	0.38
HADS anxiety	−0.58	0.04
HADS depression	−0.50	0.08
PCS	−0.52	0.07

HADS, Hospital Anxiety And Depression Scale; PCS, Pain Catastrophizing Scale.

## 4. Discussion

This study investigated the CPM capacity in individuals with chronic CRPS type 1 in comparison with HC. Overall, this study showed no difference in CPM capacity between chronic CRPS and HC, which may reflect preserved endogenous pain inhibition in individuals with CRPS. This finding is in line with another CPM study in acute CRPS,^20^ but contrasts previous findings in patients with chronic CRPS using alternative assessments of endogenous pain modulation.^38^

### 4.1. Intact pain inhibition in chronic complex regional pain syndrome

The current findings are in line with the only existing CPM study in CRPS by *Kumowski* et al. *2017*, which also demonstrated intact endogenous pain inhibition in acute CRPS. Together, these findings suggest that CPM capacity is unimpaired in both the acute and chronic stage of CRPS. However, many pathomechanisms of CRPS are dynamic and change over time, from inflammatory processes in the acute stage to maladaptive neuroplasticity in the central nervous system in the chronic stage.^4,7^ Evidence from animal studies suggests that initial inflammatory mechanisms can lead to a subsequent increase of pain inhibitory processes.^29,34^ It is, however, hypothesized that this early increase of endogenous pain inhibition, following the use of chronic constriction injury model in rats, might normalize over time which could be associated with the resolution of neuroinflammatory drivers in the affected limb.^29^ Under this assumption, one would assume a decrease in CPM capacity over time, as in the early phase of CRPS, individuals show increased inflammation of their affected limb which often resolves over time.^10,26^ Such findings were shown in individuals with neuropathic pain after spinal cord injury where CPM capacity decreased from early after the injury to a later stage.^11^ In this study, a decrease in CPM capacity with increasing disease duration was not observed. By contrast, within our chronic CRPS cohort, the CPM capacity tended (rho = −0.55, *P* = 0.053) to increase with disease duration. All evidence considered, unimpaired endogenous pain modulation in patients with acute as well as chronic CRPS could be a result of ongoing inflammation in the chronic stage,^30^ leading to increased pain inhibition. Per definition, CPM measures net efficacy of descending pain pathways, including both inhibitory and facilitatory mechanisms.^32^ Therefore, the finding of unimpaired CPM capacity in CRPS compared with HC does not negate the possibility that enhanced pain facilitatory effects could counteract increased pain inhibition.

Our findings of preserved CPM capacity in CRPS seem to contrast those of *Seifert* et al. *2009*, which used another measure of endogenous pain modulation, ie, pain adaptation. They reported a lack of pain adaptation to repetitive electrical stimulation, as well as the subsequent development of a larger area of mechanical hyperalgesia in individuals with CRPS. One possible explanation for these opposing findings could be the different approaches used to investigate endogenous pain modulation. While both pain adaptation and CPM represent aspects of endogenous pain modulation,^41^ pain adaptation was not found to be directly associated with CPM capacity in HC^46^ or in chronic pain patients (ie, low back pain and knee osteoarthritis).^42^ In addition, pain adaptation also involves peripheral processes such as fatigue of peripheral nociceptive neurons.^14^ Therefore, a direct comparison of results should be avoided. The lack of pain adaptation observed by Seifert and colleagues could possibly be explained by maladaptive pain facilitatory mechanisms.^38^ Interestingly, a previous study in an extended sample of our CRPS cohort found exaggerated temporal summation of pain in individuals with CRPS compared with HC.^37^ This would further support the notion that increased pain facilitatory mechanisms, rather than impaired pain inhibition, contribute to the pain experience in chronic CRPS. Furthermore, we corroborated previous QST studies in CRPS^8,13,33^ showing peripheral hypersensitivities toward static pressure pain and punctate mechanical pain in the affected limb of individuals with CRPS. However, this pronounced hypersensitivity in the CRPS-affected limb did not result in a net decrease of CPM capacity when tested in the painful limb. By contrast, our results show intact CPM capacity in CRPS not only when tested in the painful but also the control limb. This suggests that these pain hypersensitivities may occur independently of generalized deficits in descending pain inhibition. Interestingly, experimental studies inducing primary and secondary hyperalgesia in healthy participants showed that specifically pressure-evoked hyperalgesia can be linked to peripheral sensitization.^18,19^ Thus, the mechanical hypersensitivities in our CRPS cohort might be partially driven by peripheral facilitatory mechanisms.

Taken together, we cannot conclude on generally decreased endogenous pain inhibition in our CRPS cohort. The finding of comparable CPM capacity in individuals with CRPS and HC in this study highlights the need to explore alternative explanations for the disproportionate pain these individuals suffer from, such as peripheral impairments or central pain facilitation.

### 4.2. Clinical pain is not associated with conditioned pain modulation capacity

Overall, pain characteristics of individuals with CRPS were not related to their CPM capacity. Although some studies found associations between CPM capacity and pain duration (central neuropathic pain),^11^ pain extent (chronic back pain,^12^ central neuropathic pain),^15^ and pain intensity (chronic pancreatitis),^2^ our findings are in line with most studies,^9^ where CPM capacity was not found to be associated with clinical manifestation of pain, eg, intensity and spatial pain extent. Although previous studies report no influence of anxiety, depression, and pain catastrophizing on CPM capacity in healthy individuals as well as acute and chronic pain patients,^25,28,31^ we found that more anxious individuals with CRPS showed higher CPM capacity. This finding was unexpected and contrasts a recent study in healthy individuals showing that negative emotions reduce and positive emotions increase CPM capacity.^21^ One possible explanation could be increased stress-induced analgesia^5^ in more anxious individuals with CRPS which was also postulated to lead to decreased CPM capacity in optimists compared with pessimists.^17^ However, as the perceived level of stress during the CPM paradigm was not recorded in this study, empirical support for this assumption is lacking. Therefore, the association of psychological factors and endogenous pain modulation, both in healthy participants and chronic pain patients, warrants further investigations.

### 4.3. Limitations

The relatively small sample size limits the statistical power of our analyses and, in combination with the heterogeneity of the study population in pain location, intensity, extent, and duration, may restrict the generalizability of our findings. Furthermore, individuals with hand and foot CRPS were assessed at different body sites, influencing QST and PPT values. However, calculation of z-scores enabled the standardization and pooling of the data. Potential effects of pain medication on the CPM capacity of individuals with CRPS cannot be excluded. While we are aware of the potential influence of pain medication such as opioids on CPM capacity in chronic pain patients,^27^ the heterogeneity of medication intake did not allow a subgroup analysis.

### 4.4. Conclusion

This study extends the knowledge on endogenous pain modulation in individuals with chronic CRPS type 1. In the assessed study cohort, CPM did not clearly differ between individuals with chronic CRPS and HC. Therefore, intense pain and pronounced pain hypersensitivities in the painful limb of CRPS may not primarily result from decreased pain inhibition, but rather due to pain facilitatory mechanisms. The question whether pain inhibitory and pain facilitatory mechanisms change over the course of the disease remains open and warrants future research. In particular, longitudinal studies could shed light on to changes of pro- and antinociceptive mechanisms from the acute to the chronic stage in CRPS.

## Disclosures

The authors declare no conflicts of interest regarding this work.

## Supplemental digital content

Supplemental digital content associated with this article can be found online at http://links.lww.com/PR9/A354.
